# Zoonoses research in Somalia: A scoping review using a One Health approach

**DOI:** 10.1016/j.onehlt.2023.100626

**Published:** 2023-09-01

**Authors:** Farah I. Mumin, Andy Fenton, Abdinasir Yusuf Osman, Siobhan M. Mor

**Affiliations:** a Institute of Infection, Veterinary and Ecological Sciences, University of Liverpool, Neston, United Kingdom; b International Livestock Research Institute, Addis Ababa, Ethiopia; c Faculty of Veterinary Medicine, Red Sea University, Bosaso, Puntland State, Somalia; d Royal Veterinary College, University of London, London, United Kingdom; e National Institute of Health, Ministry of Health, Mogadishu, Somalia

**Keywords:** Zoonoses, Somalia, Research, Diseases: One Health

## Abstract

Zoonoses are likely to cause a substantial burden on both human and animal health systems in Somalia, given the close proximity between the pastoralist majority and their livestock. However, decades of instability leading to weak disease surveillance have meant that data on the burden of zoonoses is lacking. The aim of this scoping review was to assess and synthesize the available literature on the presence and burden of zoonoses in Somalia.

We used keywords to search Web of Science for relevant publications. Studies were included if they contained relevant data on a zoonosis and were undertaken in Somalia or were undertaken in another country where exposure could reasonably be assumed to have occurred in Somalia (e.g., migrants/refugees, returning soldiers, exported animals). Studies were not included if they focused on Somali ethnic communities permanently living elsewhere or if zoonotic aspects were not considered. We extracted data on disease(s) reported, geographic focus, data reported (human, animal, environment), study design and author affiliation.

A total of 22 zoonotic infections were documented in 76 publications. The most frequently studied diseases were Rift Valley Fever (*n* = 15, 17%), brucellosis (*n* = 13, 14%) and hepatitis E (*n* = 10, 11%). Around 30% of papers reported data from relevant populations outside Somalia. Only 18 papers undertook laboratory analysis within Somalia. Most papers reported data on humans (45%) and animals (36%) with limited research on the environmental domain. Descriptive studies (47%) dominated and most were led by non-Somali researchers (89% in first authors and 95% of last authors).

This study highlights the need for well-designed zoonoses research in Somalia supported by capacity building of local researchers and investments in diagnostic laboratories.

## 1 Introduction

Livestock is the main asset for pastoralists and sedentary agriculturalists of Somalia, where regional trade with the Arabian Peninsula contributes substantially to the national economy [1]. The livestock sector directly or indirectly employs 65% of the Somali population, generates 40% of the nation's Gross Domestic Product (GDP), and is a source of over 80% of the hard currency in the country [2]. Pastoralists' income is not diversified and thus trading livestock and livestock products is their only source of sustenance and livelihoods [3]. Consequently, trade bans related to Rift Valley fever (1998–2009) and COVID-19 related disruptions to the Hajj pilgrimage (which decreased trade during the peak season) [4,5] significantly impacted on household economics of pastoralists and contributed to lost tax revenue for the government.

The close association between pastoralists and their livestock increases the risk of zoonoses transmission [6]. This risk is exacerbated in Somalia by the lack of accessible health services due to the remoteness and mobility of pastoralists as well as the country's fragile health system. Most pastoralists engage in the practice of transhumance, which is the seasonal movement of grazing animals. Such transhumance creates interdependent relationships between humans and animals that are sensitive to the increased frequency of droughts brought about by climate change in Africa [7]. Moreover, the uncontrolled movement of livestock and people across borders between Somalia, Ethiopia and Kenya along with informal trade routes can facilitate the spread of zoonotic diseases across the region [8].

More than 30 years of state collapse and civil unrest in Somalia has resulted in limited surveillance and research into zoonotic diseases, except for some technical humanitarian reports that show a high burden of infectious diseases in human population [9]. There has been a gradual increase of health research output after the formation of the Federal Government of Somalia (FGS) in 2012 [10] but very few of these studies have specifically focused on zoonoses [11]. Somalia's National Development Plan prioritizes control of infectious and communicable diseases [12] but this is hindered by a lack of evidence on the zoonotic diseases prevalent in the country. Identification of priority zoonoses of national concern is recommended to help guide investments in surveillance and research [13]. However, unlike other East African countries such as Kenya, Ethiopia, and Uganda [[14], [15], [16]], no systematic zoonotic disease prioritization exercise had been supported in Somalia until recently.

We conducted a systematic review of Somalia's zoonoses research with the aim of 1) describing the major zoonotic infections reported; 2) identifying the geographic regions where research has been conducted; 3) evaluating the types of data reported (human, animal, environment) and populations under study; 4) describing the study design and methods; and 5) analyzing authorship origin and affiliations. The output from this review informed discussions held during the One Health Zoonotic Disease Prioritization workshop for Somalia, the findings of which are reported in another article in the same issue (reference to be added upon acceptance).

## 2 Methodology

This scoping review followed the guidelines presented in the Preferred Reporting Items for Systematic Reviews and Meta-Analyses (PRISMA) extension for scoping reviews (Supplementary File 1) [17].

### 2.1 Search approach

Database searching was conducted using the Web of Science (WoS) platform, which incorporates several databases (Science Citation Index, Social Sciences Citation Index, Arts and Humanities Citation Index, Conference Proceedings Citation Index, Conference Proceedings Citation Index, Social Science & Humanities Book Index, Science Book Citation Index, Social Science and Humanities, BIOSIS Citation Index, Current Contents Connect, Data Citation Index, Derwent Innovations Index, KCI Korean Journal Database, MEDLINE, SciELO Citation Index and Zoological Records). All databases and collections were included in the search. Since no lists of zoonoses present in Somalia were available, we consulted several sources to develop an initial list of disease search terms. This included the top five diseases identified as part of a (non-systematic) zoonotic diseases prioritization workshop in Somalia [18]; top 20 diseases identified in zoonoses prioritization workshops held in other East African countries (Kenya, Ethiopia and Uganda) [[14], [15], [16]] and lists developed as part of two systematic reviews on zoonoses in East Africa/Horn of Africa [11,19]. This resulted in a shortlist of 18 most-commonly cited diseases (Supplementary File 2). This initial list was supplemented with six additional diseases (*E. coli*, rickettsiosis, *Staphylococcus aureus*, Crimean-Congo hemorrhagic fever, West Nile fever and liver fluke) following a rapid search using keywords such as zoonoses AND Somalia. Somaliland is an autonomous region in northern Somalia which declared independence from Somalia in 1991. Since no similar study has been done for Somaliland, we elected to include (and specifically search for) studies from this region. The final search strategy involved combining several text strings using the basic formula:

$$\left(A\;\mathrm{OR}\;B\right)\;\mathrm{AND}\;C$$

where A was a list of specific disease/pathogen search terms (see Supplementary File 2; adapted from Cavalerie et al. [11]) combined using the Boolean operator ‘**OR**’; B were general terms designed to capture other zoonoses (Zoonoses **OR** Zoonosis **OR** Zoonotic **OR** Zoonotic diseas*); and C were geographic search terms designed to limit the scope (Somalia **OR** Somaliland **OR** Somali*). Database searching was performed on 26th July 2022. No limits were placed on date of publication or language. Retrieved references were exported to citation management software (Endnote X9.3.3; Clavariate Analytics, Philadelphia, PA) for deduplication as previously described [20].

### 2.2 Screening and data extraction

Deduplicated records were imported into systematic review software (Covidence; Veritas Health Innovation, Melbourne, Australia). Records were further deduplicated using the built-in feature in Covidence to identify any duplicates missed by Endnote. Two reviewers (FM and SM) independently screened the title and abstract of all studies, with clearly irrelevant studies excluded at this stage. Subsequently the same reviewers read the full paper to confirm whether they met the eligibility criteria (Table 1). Conflicts were resolved through discussion or by inviting a third reviewer (AF) where necessary. Studies were included if they clearly reported information on the zoonosis situation in Somalia e.g., study involving primary data collection on a zoonotic disease in populations within Somalia. Studies on diseases with both zoonotic and non-zoonotic aspects (i.e., tuberculosis, leishmaniasis, trypanosomiasis, schistosomiasis, dengue, yellow fever, chikungunya, and Zika) were closely scrutinized and excluded at this stage if they did not contain evidence of zoonosis (e.g., study on tuberculosis in humans which did not distinguish between *M. tuberculosis* and *M. bovis*). The limited amount of research originating in Somalia required us to consider the relevance of studies undertaken in other countries. This included reports on: zoonotic infections diagnosed in Somali migrants living in (non-endemic) countries; soldiers and staff deployed to Somalia and diagnosed with a zoonosis on return to a (non-endemic) country; and livestock exported from Somalia and screened for a zoonosis on arrival in the importing country. In all these cases, we considered the populations to be sentinels for the situation in Somalia and hence the studies were included, and provided evidence of the risk of zoonosis transmission through international migration [21]. In contrast, studies on Somali ethnic communities that live permanently in Ethiopia (Somali Region) and Kenya (Northeastern) were excluded as such studies were deemed to be potentially unrelated to the situation in Somalia. Studies involving diagnostic test validations and those which solely addressed antimicrobial resistance were deemed ineligible if the link to zoonoses in Somalia was tangential (e.g., study in US using banked control serum from Somali patients [22]).

**Table 1:** Inclusion and exclusion criteria for scoping review of zoonoses in Somalia.

**Inclusion criteria**	**Exclusion criteria**
• Studies with total or partial focus on one or more zoonoses (outcome) in Somalia • Studies involving strains from Somalia • Studies of migrants/refugees from Somalia • Reports on zoonotic infection of foreign staff/soldiers deployed to Somalia	• Studies not relevant to zoonoses in Somalia • Study of tuberculosis/leishmaniasis/ trypanosomiasis/schistosomiasis where zoonotic aspects are not considered • Study of dengue/yellow fever/chikungunya/Zika but no evidence of zoonotic transmission • Focus on established Somali populations in Kenya/Ethiopia • Studies not written in English language • Studies where full text was not available after searching and requesting from the library • Studies with no relevant primary data/summary statistics presented

To ease data management, data extraction was performed for selected studies using a form that was developed in KoBo Toolbox (Harvard Humanitarian Initiative, Cambridge, MA). The form captured data on year of publication, zoonotic diseases reported, geographic focus (including sub-national region and whether Somalia was partial or total focus), data reported (One Health domains – human, animal, environment – as adapted from Cavalerie et al. [11] and population characteristics), study design, and author affiliation. Data extraction was performed by one of the reviewers (FM) using KoBo Collect.

### 2.3 Data analysis

The extracted data were downloaded from the KoBo Toolbox server as a CSV file and imported into SPSS statistical software (IBM Inc.; Chicago, IL) for analysis. Data were summarized using frequency tables. A map depicting research output by region was created using the free and open source QGIS. Finally, for the most studied diseases, the qualitative/quantitative findings from individual papers were summarized.

## 3 Results

### 3.1 General findings

**Fig. 1:**
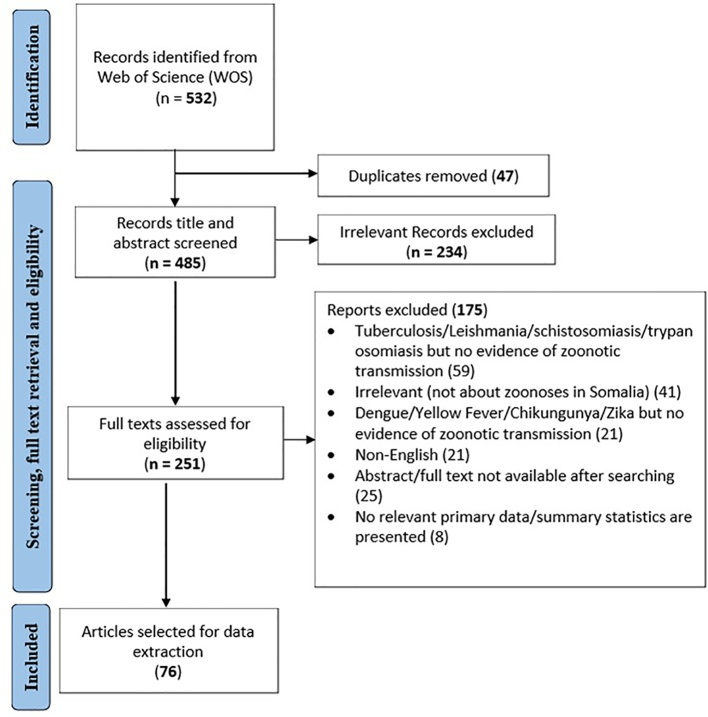
PRISMA flow diagram showing the number of publications on zoonoses in Somalia screened, reviewed against the inclusion/exclusion criteria, and selected for data extraction.

**Fig. 2:**
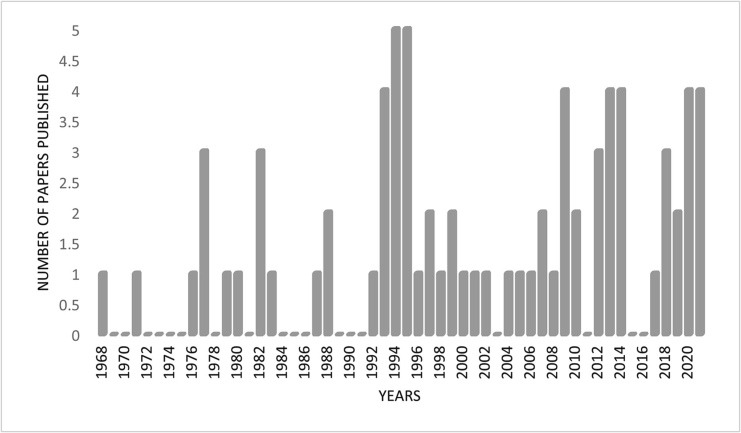
Number of publications on zoonoses in Somalia, by year of publication.

Fig. 1 shows the PRISMA flow diagram that depicts the number of records retrieved, screened, and included in the analysis. The literature search identified a total of 532 records. Following removal of 47 duplicates, the title and abstract of 485 papers was screened and 234 were excluded as being irrelevant at this stage. Of the 251 papers that proceeded to full text review, a further 175 were excluded for different reasons (Fig. 1). The final analysis included 76 papers, with a publication year ranging from 1968 to 2021 (median 2000) (Fig. 2; Supplementary File 3).

#### 3.1.1 Disease focus

Overall, 22 zoonotic diseases were reported across 76 papers. Most of the studies focused on one or more zoonotic diseases (*n* = 57, 75%) whereas the remaining studies combined zoonotic and non-zoonotic diseases. Table 2 shows the number of publications by disease. Most studies focused on Rift Valley fever (*n* = 15), brucellosis (*n* = 13), and hepatitis E (*n* = 10), followed by echinococcosis (hydatidosis) and Q fever (*n* = 6 each) as well as Crimean-Congo hemorrhagic fever (CCHF), salmonellosis and toxoplasmosis (*n* = 5 each). The remaining diseases were reported in less than 5% of studies. The key findings for each of the most well-studied diseases are presented later.

**Table 2:** Number of publications on zoonoses in Somalia, by disease (*n* = 76).

**Disease**	**Frequency**^a^	**Percentage**
Rift Valley fever	15	16.7
Brucellosis	13	14.4
Hepatitis E	10	11.1
Echinococcosis (hydatidosis)	6	6.7
Q fever	6	6.7
Crimean-Congo hemorrhagic fever	5	5.6
Salmonellosis	5	5.6
Toxoplasmosis	5	5.6
Campylobacteriosis	3	3.3
*E. coli*	3	3.3
Fascioliasis (liver fluke)	3	3.3
Rickettsiosis	3	3.3
Leptospirosis	2	2.2
Camelpox	2	2.2
Middle East respiratory syndrome (MERS)	2	2.2
Anthrax	1	1.1
Cysticercosis/taeniasis	1	1.1
Other arboviruses	1	1.1
Trypanosomiasis	1	1.1
Nagari virus (reassortment of Bunyanwera orthobunyavirus)	1	1.1
Novel *Streptococcus infantarius* subsp. Infantarius	1	1.1
*Streptococcus equi* subsp. Zooepidemicus	1	1.1

a Frequency does not add up to the total number of papers (n = 76) because some studies included more than one disease.

#### 3.1.2 Geographic focus

**Fig. 3:**
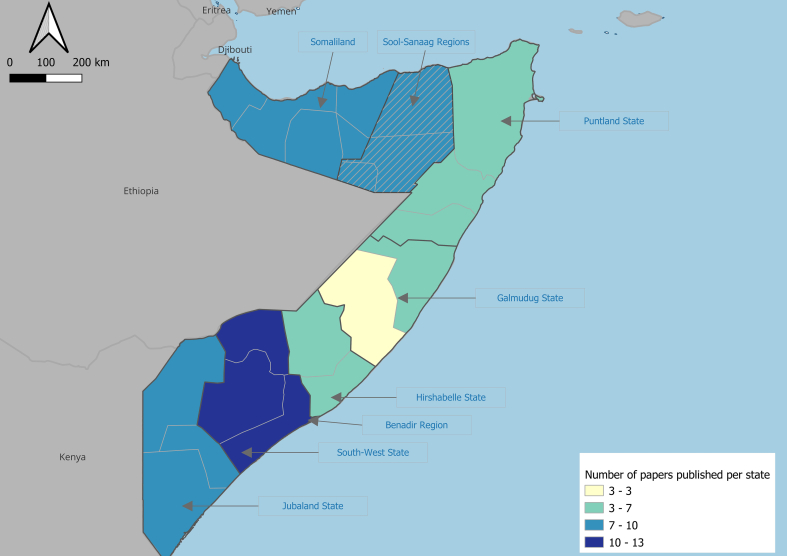
Map showing number of publications on zoonoses in Somalia, by geographic location. Figure includes 42 studies where the sub-national location within Somalia was stated. The number of studies exceeds 42 because some studies were undertaken in more than one location.

While most studies (*n* = 46, 60.5%) focused exclusively on Somalia/Somaliland, a substantial number of papers included Somalia as part of a wider study of zoonoses in the Horn of Africa region (*n* = 14, 18.5%), African continent (*n* = 8, 10.5%) or globally (n = 8, 10.5%). Table 3 shows the location of data collection and laboratory analysis. Most studies (n = 46, 60.5%) collected data from populations within Somalia however a substantial number (*n* = 22, 30%) reported data collection from relevant populations outside Somalia. Only 18 studies analyzed laboratory specimens in Somalia suggesting that most studies that collected samples in Somalia exported them for analysis. The sub-national location of studies undertaken within Somalia is shown in Fig. 3. Among 42 studies that reported the location, most were done in Benadir region (which includes the capital, Mogadishu) and Southwest State (*n* = 13, 31% each) followed by Jubaland (*n* = 9, 21%), Hirshabele (*n* = 7, 17%), Puntland (*n* = 6, 14%) and Galmudug (*n* = 3, 7%) States; 10 studies (24%) were conducted in Somaliland.

**Table 3:** Number of publications on zoonoses in Somalia, by country of data collection and laboratory analysis (*n* = 76).

**Country**	**Frequency**^a^	
	**Data collection**	**Laboratory analysis**
Somalia	46	18
United Kingdom	3	4
Egypt	3	3
Netherlands	3	3
United States	2	6
United Arab Emirates	2	2
Italy	1	4
Belgium	1	1
Germany	1	1
Oman	1	1
South Africa	1	1
Sweden	1	1
USSR	1	1
Zambia	1	1
Kenya	1	6
Not specified b	2	7
Not applicable c	13	20

a Frequency does not add up to the total number of papers (n = 76) some studies collected data and/or analyzed data in more than one country.

b Papers did not mention location of data collection or laboratory analysis.

c Papers did not involve data collection and/or laboratory analysis (e.g., mapping studies using existing data, reviews).

#### 3.1.3 One Health domains and populations under study

**Fig. 4:**
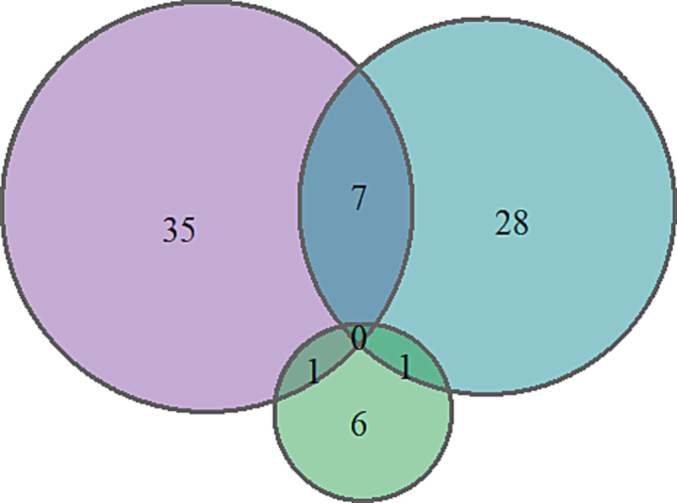
Venn diagram showing number of studies in One Health domains in Somalia. Dark violet color, dark cyan and lime green colored circles represent number of papers in human, animal and environmental domains respectively. (For interpretation of the references to color in this figure legend, the reader is referred to the web version of this article.)

Table 4 shows the number of papers by One Health domains and population under study. Most of the studies exclusively focused on humans (*n* = 34, 45%) and animals (*n* = 27, 36%) with little attention to the environment domain (*n* = 5, 7%) (Fig. 4). Few studies reported data on two or more domains simultaneously. Of 43 studies which reported data on humans, only 4 studies focused on pastoralists and no studies focused on high-risk occupations (i.e., farmers, abattoir workers, milk/meat handlers) or vulnerable groups (i.e., HIV-positive, pregnant, internally displaced persons). Of 36 studies which reported data on animals, most focused on livestock (*n* = 29, 80.5%) with few studies investigating livestock products (*n* = 3, all focused on milk). No studies investigated wildlife species.

**Table 4:** Number of publications on zoonoses in Somalia, by One Health domain and population under study (n = 76).

**One Health Domain**	**Frequency**^a^	**Population under study**	**Frequency**^a^
Human	43	General population within Somalia	15
		Somali migrant/refugee outside Somalia	9
		Hospital patients (e.g., in/out-patients)	7
		Foreign nationals deployed to Somalia (staff/soldiers)	5
		Pastoralist	4
		Not specified	8
Animal	36	Livestock species b	29
		Livestock products (meat, milk, eggs, etc.)	3
		On-host ectoparasites (fleas, ticks, lice, etc.)	3
		Companion animals	1
		Wildlife	0
		Not specified	3
Environment	8	Abiotic (e.g., weather/climate)	6
		Biotic (free-living invertebrates such as mosquitoes, ticks, mites, flies, snails)	2

a Frequency does not add up to the total number of papers (n = 76) because some studies included more than one domain and/or studied more than one population.

b Livestock category (*n* = 29) comprised camel (*n* = 18, 62%), cattle (*n* = 14, 48%), goat (n = 14, 48%), sheep (n = 10, 34%), and poultry (n = 1, 3%).

### 3.2 Study design and methods

Table 5 shows the study design and methods used in the 76 studies on zoonoses in Somalia. Descriptive epidemiological studies (e.g., prevalence survey) was the major study design employed and accounted for nearly half of the studies overall (*n* = 36, 47%). No epidemiological studies tested interventions. Serological testing was the predominant method employed in 23/36 (64%) of laboratory studies. Economic studies were infrequent (n = 3) and no studies reported using social science methods, policy analysis or programme evaluation.

**Table 5:** Number of publications on zoonoses in Somalia, by study design and methods (n = 76).

**Methods**	**Frequency**^a^	**Sub-category**	**Frequency**^a^
Epidemiology	45	Descriptive epidemiology b	36
		Observational epidemiology c	9
		Experimental epidemiology	0
		Participatory epidemiology	0
		Mathematical or advanced statistical modelling (e.g., SIR, network analysis)	0
Laboratory	36	Serology	23
		Molecular (e.g., PCR detection, sequencing)	9
		Microbiology/parasitology (e.g., bacterial/viral culture of clinical specimens, stool examination)	5
		Drug resistance (AMR)	2
		Histopathology (e.g., examination of tissues under microscope)	0
		Plant pharmacology (e.g., testing of plants for activity against pathogens)	0
		In-vitro studies (e.g., producing/testing vaccine in cell culture)	0
		In-vivo studies (i.e., animal study)	0
Review	11	Narrative review	9
		Systematic review with meta-analysis	1
		Systematic review without meta-analysis	1
Environmental health and environmental science	6	Ecological/spatial modelling of infectious agent/host/vector populations	5
		Entomology	1
Other	3	Economics (e.g., economic impact, cost-effectiveness)	3
		Social science	0
		Policy analysis	0
		Programme evaluation	0

a Frequency does not add up to the total number of papers (n = 76) because some studies included more than one method.

b Includes case reports/case series, prevalence/incidence survey, outbreak report, mapping i.e., studies which describe cases by person-animal/place/time, do not identify or quantify risk factors.

c Includes cross-sectional, case-control, cohort i.e., studies which identify and quantify risk factors, test hypotheses.

#### 3.2.1 Author affiliations

Table 6 shows affiliations of first and last authors of all studies included. Most studies (*n* = 54, 71%) were led by researchers with affiliations from high-income countries. Only a quarter (*n* = 19, 25%) of papers had at least one author with local/Somali affiliation included anywhere in the authorship and only two studies [23,24] were authored exclusively by people with a local affiliation, namely Somalia's former Serum and Vaccine Institute (SVI).

**Table 6:** Number of publications on zoonoses in Somalia, by first and last author affiliations (n = 76).

**Country**	**Frequency (%)**^a^	
	**First author affiliation**	**Last author affiliation**
United States	18 (23.7)	16 (21.1)
Somalia	8 (10.5)	4 (5.3)
Italy	7 (9.2)	6 (7.9)
United Kingdom	6 (7.9)	7 (9.2)
Another high-income country	23 (30.3)	25 (32.9)
Another low-income country	12 (15.8)	12 (15.8)

a Frequency does not add up to the total number of papers (n = 76) because some studies did not state the author affiliation (n = 2), were authored only by one person (n = 4), or authors had a duel affiliation (n = 1).

### 3.3 Disease specific findings

#### 3.3.1 Rift Valley fever (RVF)

Rift Valley fever (RVF) was the most commonly studied disease accounting for 17% (*n* = 15, 19.7%) of all studies on zoonoses in Somalia. These studies reported findings from RVF outbreaks in Jubaland and Southwest states in 1997–1998 and 2006–2007 [[25], [26], [27], [28]] and quantified the economic impact of resultant livestock export bans on Somalia [4,5,29]. One study described the results of serological surveys undertaken as part of active surveillance in Somaliland and Puntland between 2001 and 2004; animal-level prevalence ranged between 2 and 15% depending on location and year (*n* = 9517 animals sampled) [30]. More recently, in 2021, a survey of ruminants in Southwest State found a seroprevalence of only 0.3% (*n* = 609 animals sampled) [31]. Several regional studies used climate and remote sensing data to predict RVF outbreaks using risk mapping approaches [[32], [33], [34]]. A study of the Eastern Mediterranean region which compiled case data on viral hemorrhagic fevers (VHFs) assessed Somalia as being ‘medium affected’ for VHFs, based on a reported 27,500 cases and 135 human deaths from RVF from 1995 to 2015 [35].

#### 3.3.2 Brucellosis

Brucellosis was the second most studied zoonosis in Somalia accounting for around 15% (*n* = 13, 17.1%) of studies. These studies employed different testing modalities making comparison across species, time, and space difficult. Surveys undertaken in Southwest State and Benadir region in the 1970s reported seroprevalence in government farms, pastoralist livestock, and humans of 2.7%, 11.9% and 0.6%, respectively (slow agglutination microtiter method; [23]). Studies in cattle in Benadir and Jubaland States reported a seroprevalence of 9.5% (Milk Ring Test, MRT), 12.2% (Serum Agglutination Test, SAT) and 47% (Compliment Fixation Test, CFT), respectively [24]. Another serological investigation on goats slaughtered in Mogadishu abattoir reported a seroprevalence of 2.8% (Rose Bengal Plate Test, RBPT), 2.8% (SAT), 1.6% (2-Mercaptoethanol test), and 5.6% (Coombs Antiglobulin Test, AGT), respectively [36]. In the 1980s, studies of camels, cattle, sheep and goats in Benadir and Jubaland States reported a seroprevalence of 10.4%, 15.5%, 7.2% and 5.2%, respectively (SAT; [37]). Studies conducted in the 1990s found that seroprevalence in camels in Galmudug and Hirshabele States was 1.9% and 0.3%, respectively (SAT and CFT; [38]). Since 2009, surveys of camels in Puntland State and Somaliland reported 7% and 3.1% seropositivity, respectively (competitive and indirect enzyme-linked immunosorbent assay (ELISA); [39,40]) while surveys of cattle, sheep and goats in Southwest State found a seroprevalence of 19.4%, 4.4% and 7.9%, respectively (modified RBPT; [31]). Two studies were case reports of Somali migrants to the UK who were later confirmed to have brucellosis [41,42].

#### 3.3.3 Hepatitis E

Around 11% of studies reported on hepatitis E and none discussed a potential zoonotic reservoir. Most of the studies on hepatitis E were health investigations on foreign staff and soldiers deployed to or returned from Somalia during the civil war with seroprevalence ranging from 2% to 67% [[43], [44], [45], [46]]. Three studies reported a high prevalence of anti-hepatitis E virus antibodies (ranging from 52% to 94%) in patients with clinical hepatitis following an epidemic in Southwest State in 1988–1989 [[47], [48], [49]]. A study in Italy also reported positive serology in recent arrivals from Somalia (refugees), some of whom had evidence of recent infection (IgM antibodies) [50].

#### 3.3.4 Echinococcosis (hydatidosis)

Studies in the 1960s reported echinococcosis seroprevalence in humans, camels, cattle and goats of 4.2%, 31%, 67% and 50%, respectively [51,52]. Somali livestock exported to Egypt (cattle, method of detection not stated; [51]) and Oman (2.5% of goats on postmortem inspection; [53]) were also reported to be infected by *Echinococcus granulosus* and cystic hydatidosis, respectively. Two studies reported the diagnosis and removal of hydatid cysts in Somali patients in Somaliland and United States [54,55].

#### 3.3.5 Q fever

A narrative review of Q fever in North Africa and the Near and Middle East suggested that Somalia is at high risk for Q fever considering it has the largest camel population worldwide and it is customary to drink raw milk [56]. A retrospective study in Somaliland revealed a Q fever seroprevalence of 37% in adult humans [57]. Similarly, another survey of Somali refugees in Kenya found 25% seropositivity in this population [58]. Most recently, *Coxiella burnetii* was detected in 59% of ticks collected from camels in Puntland State, which supports the assertion that Q fever is endemic in the country [59].

#### 3.3.6 Crimean-Congo hemorrhagic fever (CCHF)

Serological surveys and molecular investigations of ticks obtained from livestock imported to the United Arab Emirates and Egypt in the 1990s both revealed CCHF infection of Somali livestock [60,61]. More recently, a continent-wide modelling study of viral hemorrhagic fever emergence and spread found that Somalia was particularly vulnerable to ongoing transmission of CCHF following an index case due to population susceptibility and poor response capacity [62].

#### 3.3.7 Salmonellosis

A study in the 1970s claimed *Salmonella cholerae-suis* was the major cause of a camel outbreak in Somaliland after other suspected causes, such as pasteurellosis and anthrax, were ruled out [63]. Another study undertaken around the same time isolated 54 *Salmonella* strains from animal and environmental samples demonstrating the diversity of circulating stains at that time [64]. Another study reported the isolation of *Salmonella* group E from a foreign soldier in Somalia in the 1990s [65].

#### 3.3.8 Toxoplasmosis

Serosurveys conducted in the 1980s on *T. gondii* in humans and animals reported a seroprevalence of 38% and 56% in Southwest State, 40% and 43% in Benadir region, and 10% and 39% in Hirshabele State, respectively [52,[66], [67], [68]].

## 4 Discussion

We previously showed that research on zoonoses is highly limited in Somalia compared to other countries in the Horn of Africa region [11]. In this paper we build on this finding by collating and analyzing the published research on zoonoses in Somalia in detail, giving comprehensive insight on the literature gaps across the pre- and post-civil war periods. Although the risk of zoonoses transmission in Somalia is thought to be high due to the population's reliance on animal husbandry, as well as poor slaughterhouse hygiene and the cultural practice of drinking raw milk, little attention has been given to the study of zoonotic diseases in the country. We show that this research has been limited to just 22 zoonotic diseases. Most of the studies are descriptive and have not involved multi-disciplinary data collection. Studies have not targeted populations at highest risk for zoonoses and have largely been conducted by non-local researchers.

The dominance of descriptive studies such as case reports and prevalence surveys provide a low level of evidence for the burden of zoonoses in Somalia. This finding is not unique; previous scoping reviews in the Horn of Africa have made similar findings across countries [11,19]. Disease descriptions are essential for understanding the disease status of a country. It is however notable that insights into the epidemiological situation in Somalia have often been learned through study of non-resident populations, such as exported animals, soldiers and migrants. Millions of Somalis fled the country due to the collapse of the central government in 1991 and subsequent civil war [69]. Health screening of refugees on arrival in their new host country proved a significant source of information in the current study, as did screening of soldiers posted to Somalia. Although the findings from these case series cannot be extended to the general population, in the absence of other data, they do provide important evidence on the presence of specific zoonoses within Somalia. Future studies should target populations at high risk for zoonoses, such as pastoralists, as well as vulnerable groups, such as internally displaced populations. Many such people are former pastoralists who have been forced to abandon their livelihood due to conflict or following death of their livestock due to prolonged drought [70]. The need for research on zoonotic infections in the context of internal displacement has been noted by others [71].

The research attention directed at RVF is consistent with the significant economic impact of this disease on the country. In Somaliland (southern Somalia) alone, the ban imposed by Saudi Arabia in 1998 has costed $330 million to livestock exporters [72]. For this reason, RVF has been targeted for research [11] and ranked highly in zoonoses prioritization exercises [[14], [15], [16]] throughout the region. Similarly, brucellosis has been the focus of much zoonosis research in the Horn of Africa [11]. The research focus on hepatitis E is regionally novel and likely reflects the vulnerability of internally displaced populations to outbreaks [73]. Hepatitis E is now regarded as an emerging zoonosis [74], although no evidence of zoonotic transmission was reported in the studies in Somalia.

The limited authorship contribution and/or acknowledgment of local researchers likely reflects broader trends in academic publishing where African researchers are under-represented [75] as well as limited local diagnostic and research capacity in Somalia. A baseline assessment after the civil war identified weak governance in the higher education system, financial hardship, and lack of capacity building opportunities among the factors hampering Somalia's research output [76]. The lack of local diagnostic capacity has often resulted in the transportation of biological samples outside of the country for analysis. The WHO's International Health Regulation report highlighted that samples are usually transported to Kenya and beyond for analysis [18]. Lack of laboratory capacity was further exposed during the COVID-19 pandemic where samples were transported to neighboring countries for molecular analysis [77]. In addition, the departure of local researchers during the war and the collapse of government institutions played a role in research marginalization of Somalia. Consequently, the country became disconnected from regional and continental initiatives that funded One Health research and networking programs such as USAID’ Emerging Pandemic Threats Program [78]. Capacity building of local researchers and an increase in funding and collaborations can aid in Somalia's health system recovery and increase research output designed and led by local researchers [79].

There are several methodological limitations to this study. Database searching was limited to the WoS platform and did not extend to grey literature. While WoS hosts the largest congregate of databases (including MEDLINE/PubMed) our review may have missed some papers indexed elsewhere. Search terms were constructed using names of zoonoses that have been prioritized in the region. Whilst this approach likely identified the diseases of most importance, it is possible that papers on other diseases were missed. Furthermore, a considerable number of articles were written in non-English or were not available in full text format. Omission of these papers may have also resulted in diseases being missed. Finally, we used Somalia's current administrative zones to aggregate and map research output which may have resulted in misclassification of some papers which referred to the 18 regions system. These limitations notwithstanding, this scoping review presents the most comprehensive report on the status of zoonoses research in Somalia to date and provides a blueprint for further research investment.

## 5 Conclusion

This scoping review identified a total of 22 zoonotic diseases in Somalia, reported in 76 papers. Studies conducted to date have been largely descriptive in nature and have been led by overseas researchers with significant emphasis on non-resident populations. To enrich the local evidence on zoonoses, future studies should aim to investigate multiple One Health domains simultaneously, and target high risk populations, such as pastoralists, as well as vulnerable groups, such as internally displaced populations. Availing training, mentoring, and funding to local researchers can help strengthen their capacity to conduct research. Improvement of laboratory infrastructure will be essential for sustaining a locally-led research agenda on zoonoses into the future. In the meantime, foreign researchers should seek opportunities to support local scientists to travel abroad and gain skills when analysis of samples originating from Somalia is being undertaken.

## Declaration of Competing Interest

The authors declare no personal or financial conflict of interest.

## Data Availability

Data will be made available on request.

## Appendix A Supplementary data

Preferred Reporting Items for Systematic Reviews and Meta-Analyses (PRISMA) extension for scoping reviews checklist. Supplementary material 1

Important zoonotic diseases in the region: This table shows the list of the zoonoses identified in various sources along with the most frequently named diseases across all lists. Supplementary material 2

List of papers (n = 76) included in the study with their references. Supplementary material 3

## References

[1] African Union – Interafrican Bureau for Animal Resources (2015). The Contribution of Livestock to the Somali Economy. https://www.au-ibar.org/sites/default/files/2020-11/20160610_final_report_contribution_livestock_somalia_gdp_en.pdf.

[2] Food and Agriculture Organization of the United Nations (2012).

[3] Aklilu Y., Catley A. (2010).

[4] Mtimet N. (2021). Zoonotic diseases and the COVID-19 pandemic: economic impacts on Somaliland’s livestock exports to Saudi Arabia. Global Food Security.

[5] Soumare B. (2006). Effects of livestock import bans imposed by Saudi Arabia on Somaliland for sanitary reasons related to Rift Valley fever. Outlook Agric..

[6] World Health Organization (2009).

[7] McGuirk E.F., Nunn N. (2020).

[8] Mahmoud H.A. (2010).

[9] World Health Organization (2022). Somalia Health Cluster. https://healthcluster.who.int/countries-and-regions/somalia.

[10] Sweileh W.M. (2020). Health-related publications on people living in fragile states in the alert zone: a bibliometric analysis. Int. J. Ment. Heal. Syst..

[11] Cavalerie L. (2021). One hundred years of zoonoses research in the horn of Africa: a scoping review. PLoS Negl. Trop. Dis..

[12] Ministry of Planning Investment and Economic Development (2020).

[13] Salyer S.J. (2017). Prioritizing Zoonoses for Global Health capacity building-themes from one health zoonotic disease workshops in 7 countries, 2014-2016. Emerg. Infect. Dis..

[14] Munyua P. (2016). Prioritization of zoonotic diseases in Kenya, 2015. PLoS One.

[15] Pieracci E.G. (2016). Prioritizing zoonotic diseases in Ethiopia using a One Health approach. One Health.

[16] Sekamatte M. (2018). Multisectoral prioritization of zoonotic diseases in Uganda, 2017: a One Health perspective. PLoS One.

[17] Tricco A.C. (2018). PRISMA extension for scoping reviews (PRISMA-ScR): checklist and explanation. Ann. Intern. Med..

[18] World Health Organization (2016).

[19] Kemunto N. (2018). Zoonotic disease research in East Africa. BMC Infect. Dis..

[20] Bramer W.M. (2016). De-duplication of database search results for systematic reviews in EndNote. J. Med. Libr. Assoc. JMLA.

[21] Mavroidi N. (2008). Transmission of zoonoses through immigration and tourism. Vet. Ital..

[22] Müller M.A. (2014). MERS coronavirus neutralizing antibodies in camels, eastern Africa, 1983–1997. Emerg. Infect. Dis..

[23] Hussein A.S., Singh S.S., Haji H. (1978). A survey of bovine brucellosis in the southern parts of Somalia a comparative study of prevalence of the disease in farm animals and animals from nomadic herds. Bull. Anim. Health Product. Africa.

[24] Wernery U., Karani A., Vertal P. (1976). *Bovine brucellosis in the southern regions of the Somali Democratic Republic.* Bulletin of animal health and production in Africa. Bulletin des sante et production animales en Afrique.

[25] Outbreaks of Rift Valley fever in Kenya (2007). Somalia and United Republic of Tanzania, December 2006-April 2007. Releve epidemiologique hebdomadaire.

[26] Centers for Disease, C. and Prevention (1998). *Rift Valley Fever--East Africa,* 1997-1998. MMWR. Morbidity Mortal. Weekly Rep..

[27] Faye B. (2019).

[28] Nderitu L. (2011). Sequential Rift Valley fever outbreaks in eastern Africa caused by multiple lineages of the virus. J. Infect. Dis..

[29] Knight-Jones T.J.D. (2014). Risk assessment and cost-effectiveness of animal health certification methods for livestock export in Somalia. Prevent. Veterin. Med..

[30] Soumare B. (2007). Screening for Rift Valley fever infection in northern Somalia: a GIS based survey method to overcome the lack of sampling frame. Vet. Microbiol..

[31] Hassan-Kadle A.A. (2021). Rift Valley fever and Brucella spp. in ruminants, Somalia. BMC Vet. Res..

[32] Anyamba A. (2009). Prediction of a Rift Valley fever outbreak. Proc. Natl. Acad. Sci. U. S. A..

[33] Anyamba A. (2010). Prediction assessment of the Rift Valley fever activity in east and southern Africa 2006-2008 and possible vector control strategies. Am. J. Trop. Med. Hyg..

[34] Campbell L.P. (2019). Predicting abundances of Aedes mcintoshi, a primary Rift Valley fever virus mosquito vector. PLoS One.

[35] Altmann M., Nahapetyan K., Asghar H. (2018). Identifying hotspots of viral haemorrhagic fevers in the Eastern Mediterranean Region: perspectives for the emerging and dangerous pathogens laboratory network. East Mediterr. Health J..

[36] Falade S.H., Brucella A.H. (1979). Sero-activity in Somali goats. Trop. Anim. Health Prod..

[37] Andreani E. (1982). Serological and bacteriological investigation on Brucellosis in Domistic ruminants of the Somali Democratic - Republic. Revue D Elevage Et De Medecine Veterinaire Des Pays Tropicaux.

[38] Baumann M.P.O., Zessin K.H. (1992). Productivity and health of camels (Camelus-Dromedarius) in Somalia - associations with trypanosomiasis and brucellosis. Trop. Anim. Health Prod..

[39] Ghanem Y.M. (2009). Seroprevalence of camel brucellosis (Camelus dromedarius) in Somaliland. Trop. Anim. Health Prod..

[40] Mohamud A.S. (2021). Seroprevalence and risk factors associated with Brucella infection in camels in the Puntland State of Somalia. Veterin. Sci..

[41] Javaid M.R., Farrugia M., Noeman Ahmed Y. (2013).

[42] Wheat P.F., Dabbs D.J., Thickett K.J. (1995). Brucella melitensis: an unexpected isolate from cerebrospinal fluid. Commun. Dis. Rep. CDR Rev..

[43] Buisson Y. (1994). Hepatitis -E virus-infection in soldiers sent to epidemic regions. Lancet.

[44] Burans J.P. (1994). Threat of hepatitis -E virus infection in Somalia during operation restore hope. Clin. Infect. Dis..

[45] Sharp (1995). Illness in journalists and relief workers involved in international humanitarian assistance efforts in Somalia, 1992-93. J. Travel Med..

[46] Vandenvelde C. (1994). Hepatitis-E virus-infection in belgian soldiers. Lancet.

[47] Bile K. (1994). Contrasting roles of Rivers and Wells as sources of drinking water on attach and fatality rates in a hepatitis E epidemic in Somalia. Am. J. Trop. Med. Hyg..

[48] Chau K.H. (1993). Detection of IgA class antibody to hepatitis -E virus in serum samples from patients with hepatitis -E virus infection. J. Med. Virol..

[49] Mushahwar I.K. (1993). Serological studies of an Enterically transmitted non-a, non-B-hepatitis in Somalia. J. Med. Virol..

[50] Scotto G. (2013). Prevalence of antibodies to hepatitis E virus in immigrants: a seroepidemiological survey in the district of Foggia (Apulia-southern Italy). J. Med. Virol..

[51] Cahill K.M. (1971). Studies in Somalia. Trans. R. Soc. Trop. Med. Hyg..

[52] Kagan I.G., Cahill K.M. (1968). Parasitic serologic studies in Somaliland. Am. J. Trop. Med. Hyg..

[53] Al-Kitani F. (2014). Cystic hydatidosis in slaughtered goats from various municipal abattoirs in Oman. Trop. Anim. Health Prod..

[54] Babady N.E. (2009). A 48-year-old Somali woman with hip pain. Clin. Infect. Dis..

[55] Ewnte B. (2020). Hydatid cyst of the foot: a case report. J. Med. Case Rep..

[56] Devaux C.A. (2020). *Coxiella burnetii in dromedary camels (Camelus dromedarius): a possible threat for humans and livestock in North Africa and the near and Middle East?* Frontiers in veterinary. Science.

[57] Botros B.A.M. (1995). Coxiella-Burnetii antibody Prevalences among human-populations in NorthEastern Africa determined by enzyme-immunoassay. J. Trop. Med. Hygiene.

[58] Gray G.C. (1995). Serological evidence of respiratory and Rickettsial infections among Somali refugees. Am. J. Trop. Med. Hyg..

[59] Frangoulidis D. (2021). High prevalence and new genotype of Coxiella burnetii in ticks infesting camels in Somalia. Pathogens.

[60] Khan A.S. (1997). An outbreak of Crimean-Congo hemorrhagic fever in the United Arab Emirates, 1994-1995. Am. J. Trop. Med. Hyg..

[61] Rodriguez L.L. (1997). Molecular investigation of a multisource outbreak of Crimean-Congo hemorrhagic fever in the United Arab Emirates. Am. J. Trop. Med. Hyg..

[62] Pigott D.M. (2017). Local, national, and regional viral haemorrhagic fever pandemic potential in Africa: a multistage analysis. Lancet.

[63] Cheyne I.A., Pegram R.G., Cartwright C.F. (1977). Outbreak of salmonellosis in camels in NorthEast of Somalia Democratic Republic. Trop. Anim. Health Prod..

[64] Wernery U., Thimm B. (1978). Isolation of 54 salmonella strains including 14 serotypes from domestic-animals in Somali-democratic-republic. Zentralblatt Fur Veterinarmedizin Reihe B-Journal of Veterinary Medicine Series B-Infectious Diseases Immunology Food Hygiene Veterinary Public Health.

[65] Sharp T.W. (1995). Diarrheal-disease among military-personnel during operation-restore-hope, Somalia, 1992-1993. Am. J. Trop. Med. Hyg..

[66] Ahmed H.J. (1988). Human toxoplasmosis in Somalia - prevalence of toxoplasma antibodies in a village in the lower Scebelli region and in Mogadishu. Trans. R. Soc. Trop. Med. Hyg..

[67] Ilardi I. (1987). Epidemiologic-study of parasitic infections in Somali nomads. Trans. R. Soc. Trop. Med. Hyg..

[68] Zardi O. (1980). Serological survey of toxoplasmosis in Somalia. Trans. R. Soc. Trop. Med. Hyg..

[69] Gundel J. (2002). The migration–development nexus: Somalia case study. Int. Migr..

[70] Maxwell D., Fitzpatrick M. (2012). The 2011 Somalia famine: context, causes, and complications. Global Food Security.

[71] Braam D.H., Jephcott F.L., Wood J.L.N. (2021). Identifying the research gap of zoonotic disease in displacement: a systematic review. Global Health Res. Policy.

[72] Soumaré B. (2006). Effects of livestock import bans imposed by Saudi Arabia on Somaliland for sanitary reasons related to Rift Valley fever. Outlook Agric..

[73] Desai A.N. (2022). Viral hepatitis E outbreaks in refugees and internally displaced populations, sub-Saharan Africa, 2010-2020. Emerg. Infect. Dis..

[74] Park W.J. (2016). Hepatitis E virus as an emerging zoonotic pathogen. J. Vet. Sci..

[75] Okeke I.N. (2010). African researchers underrepresented. Science.

[76] Pellini A. (2020).

[77] Farah A.A. (2022). Double burden on health services in Somalia due to COVID-19 and conflict. Ann. Med. Surg..

[78] Killewo J., Bazeyo W., Mdegela R. (2017). One Health Central and Eastern Africa: historical and future perspectives. International Encyclopedia of Public Health..

[79] Dalmar A.A. (2017). Rebuilding research capacity in fragile states: the case of a Somali–Swedish global health initiative: Somali–Swedish Action Group for Health Research and Development. Glob. Health Action.

