# Supplementary material for: Zoonoses research in Somalia: A scoping review using a One Health approach
Source: One Health. 2023 Sep 1;17:100626. doi: 10.1016/j.onehlt.2023.100626 (PMC10665144; doi:10.1016/j.onehlt.2023.100626)
Supplement: Supplementary file 1 — Preferred Reporting Items for Systematic Reviews and Meta-Analyses (PRISMA) extension for scoping reviews checklist. [file mmc1.docx]

| Supplementary file 01 Table. Preferred Reporting Items for Systematic reviews and Meta-Analyses extension for Scoping Reviews (PRISMA-ScR) Checklist (adapted from Tricco *et al.* 2018) | | | |
| --- | --- | --- | --- |
| **Section** | **Item** | **PRISMA-ScR checklist item** | **Reported on page #** |
| **TITLE** | | | |
| Title | 1 | Identify the report as a scoping review. | See Title |
|  |  | “Zoonoses Research in Somalia: A Scoping Review Using a One Health Approach” |  |
| **ABSTRACT** | | | |
| Unstructured summary | 2 | Provide an unstructured summary that includes (as applicable): background, objectives, eligibility criteria, sources of evidence, results, and conclusions that relate to the review questions and objectives. | See abstract |
| **INTRODUCTION** | | | |
| Rationale | 3 | Describe the rationale for the review in the context of what is already known. Explain why the review questions/objectives lend themselves to a scoping review approach. | See Introduction |
| Objectives | 4 | Provide an explicit statement of the questions and objectives being addressed with reference to their key elements (e.g., population or participants, concepts, and context) or other relevant key elements used to conceptualize the review questions and/or objectives. | See introduction |
|  |  | This review aimed to address the following research questions:   1. What are the major zoonotic infections reported in Somalia? 2. What are the geographic regions where zoonoses research has been conducted? 3. What are the types of data reported (human, animal, environment) and populations studied? 4. What were the study designs and methods used? 5. Who are the authors and what are their affiliations? |  |
| **METHODS** | | | |
| Protocol and registration | 5 | Indicate whether a review protocol exists; state if and where it can be accessed (e.g., a Web address); and if available, provide registration information, including the registration number. | N/A |
| Eligibility criteria | 6 | Specify characteristics of the sources of evidence used as eligibility criteria (e.g., years considered, language, and publication status), and provide a rationale. | See methods, screening and data extraction |
|  |  | Web of Science database search was performed on 27^th^ July 2022. Studies were included if they contained relevant data on a zoonosis and (a) were undertaken in Somalia; or (b) were undertaken in another country where the exposure could be reasonably assumed to have occurred in Somalia (e.g., migrants/refugees, returning soldiers, exported animals) |  |
| Information sources | 7 | Describe all information sources in the search (e.g., databases with dates of coverage and contact with authors to identify additional sources), as well as the date the most recent search was executed. | See Methods, search approach |
|  |  | Database searching was conducted using the Web of Science (WoS) platform, which incorporates several databases (Science Citation Index, Social Sciences Citation Index, Arts and Humanities Citation Index, Conference Proceedings Citation Index, Conference Proceedings Citation Index, Social Science & Humanities Book Index, Science Book Citation Index, Social Science and Humanities, BIOSIS Citation Index, Current Contents Connect, Data Citation Index, Derwent Innovations Index, KCI Korean Journal Database, MEDLINE, SciELO Citation Index and Zoological Records) |  |
| Search | 8 | Present the full electronic search strategy for at least 1 database, including any limits used, such that it could be repeated. | See Methods, 2.1. search approach, Supplementary file 2 and limitations in the discussion |
| Selection of sources of evidence | 9 | State the process for selecting sources of evidence (i.e., screening and eligibility) included in the scoping review. | See methods, screening and data extraction section |
| Data charting process | 10 | Describe the methods of charting data from the included sources of evidence (e.g., calibrated forms or forms that have been tested by the team before their use, and whether data charting was done independently or in duplicate) and any processes for obtaining and confirming data from investigators. | See Methods, data analysis section |
| Data items | 11 | List and define all variables for which data were sought and any assumptions and simplifications made. | See Methods, screening and data extraction sections, and supplementary file 2 |
|  |  | Data extracted on disease, geographic focus, one health domain, type of data, authorship, affiliation, and method |  |
| Critical appraisal of individual sources of evidence | 12 | If done, provide a rationale for conducting a critical appraisal of included sources of evidence; describe the methods used and how this information was used in any data synthesis (if appropriate). | N/A |
| Synthesis of results | 13 | Describe the methods of handling and summarizing the data that were charted. | See Methods, data analysis section |
| **RESULTS** | | | |
| Selection of sources of evidence | 14 | Give numbers of sources of evidence screened, assessed for eligibility, and included in the review, with reasons for exclusions at each stage, ideally using a flow diagram. | See Figure 1 |
| Characteristics of sources of evidence | 15 | For each source of evidence, present characteristics for which data were charted and provide the citations. | See Figure 1 |
|  |  | All online sources were merged for analysis |  |
| Critical appraisal within sources of evidence | 16 | If done, present data on critical appraisal of included sources of evidence (see item 12). | N/A |
| Results of individual sources of evidence | 17 | For each included source of evidence, present the relevant data that were charted that relate to the review questions and objectives. | See Figures 2 and 3 and Tables 1-6 |
| Synthesis of results | 18 | Summarize and/or present the charting results as they relate to the review questions and objectives. | See Figures 2 and 3 and Tables 1-6 |
| **DISCUSSION** | | | |
| Summary of evidence | 19 | Summarize the main results (including an overview of concepts, themes, and types of evidence available), link to the review questions and objectives, and consider the relevance to key groups. | See Discussion |
| Limitations | 20 | Discuss the limitations of the scoping review process. | See Discussion |
| Conclusions | 21 | Provide a general interpretation of the results with respect to the review questions and objectives, as well as potential implications and/or next steps. | See Discussion and conclusion |
| **FUNDING** | | | |
| Funding | 22 | Describe sources of funding for the included sources of evidence, as well as sources of funding for the scoping review. Describe the role of the funders of the scoping review. | See the acknowledgment section |

*From:* Tricco AC, Lillie E, Zarin W, O'Brien KK, Colquhoun H, Levac D, et al. PRISMA Extension for Scoping Reviews (PRISMAScR): Checklist and Explanation. Ann Intern Med. 2018;169:467–473. [doi: 10.7326/M18-0850](http://annals.org/aim/fullarticle/2700389/prisma-extension-scoping-reviews-prisma-scr-checklist-explanation)
