# Supplementary material for: Zoonoses research in Somalia: A scoping review using a One Health approach
Source: One Health. 2023 Sep 1;17:100626. doi: 10.1016/j.onehlt.2023.100626 (PMC10665144; doi:10.1016/j.onehlt.2023.100626)
Supplement: Supplementary file 2 — Important zoonotic diseases in the region: This table shows the list of the zoonoses identified in various sources along with the most frequently named diseases across all lists. [file mmc2.docx]

| **Important zoonotic diseases in the region:** The below table shows the list of the zoonoses identified in various sources along with the most frequently named diseases across all lists. | | | | | | | |
| --- | --- | --- | --- | --- | --- | --- | --- |
|  | **Informal prioritization Somalia (WHO, 2016)** | **OHZDP Ethiopia (Pieracci *et al.*, 2016)** | **OHZDP Kenya (Munyua *et al.*, 2016)** | **OHZDP Uganda (Sekamatte *et al.*, 2018)** | **Systematic Review East Africa (Kemunto *et al.*, 2018)** | **Systematic Review Horn of Africa**  **(Cavalerie *et al.*, 2021)** | **Most frequently named diseases** |
| 1 | Anthrax | Rabies | Anthrax | Anthrax | Trypanosomiasis | Brucellosis | Anthrax |
| 2 | Brucellosis | Echinococcus | Trypanosomiasis | Zoonotic influenza viruses | Brucellosis | Echinococcosis | Brucellosis |
| 3 | Bovine TB | Anthrax | Rabies | Ebola viruses | Rift Valley Fever | Gastrointestinal bacteria | Bovine Tuberculosis |
| 4 | Rift Valley Fever | Brucellosis | Brucellosis | Brucellosis | Rabies | Tuberculosis | Rift Valley Fever |
| 5 | Toxoplasmosis | Leptospirosis | Rift Valley fever | Rift Valley fever (RVF) | Echinococcosis | Rift Valley fever | Rabies |
| 6 |  | Q fever | Echinococcosis (Hydatidosis) | African trypanosomiasis | Cryptococcosis | Trypanosomiasis | Echinococcosis (Hydatidosis) |
| 7 |  | Salmonella | Non Typhi Salmonellosis | Plague | Campylobacteriosis | Toxoplasmosis | Q fever |
| 8 |  | Mycobacterium bovis | Q fever | Crimean Congo hemorrhagic fever (CCHF) | Bovine tuberculosis | Rabies | Salmonella |
| 9 |  | Tularemia | Mycobacterium spps | Rabies | Dengue | Cysticercosis/Taeniasis | Cysticercosis/Taeniasis |
| 10 |  | Leishmania | Influenza and pandemics | Marburg | Ebola | Hepatitis E | Toxoplasma |
| 11 |  | Cysticercosis/Taeniasis | Cysticercosis | Salmonellosis | Leptospirosis | Leishmaniasis | Schistosomiasis |
| 12 |  | Toxoplasma | Dengue | Q-fever | Q fever | Anthrax | Zoonotic influenza viruses |
| 13 |  | Listeria | Leptospirosis | Listeriosis | Anthrax | Leptospirosis | Trypanosomiasis |
| 14 |  | Schistosoma | Schistosomiasis | Leptospirosis | Marburg | Q Fever | Leptospirosis |
| 15 |  | Avian Influenza | Yellow fever | Zoonotic tuberculosis | Schistosomiasis |  | Leishmaniasis |
| 16 |  | Campylobacter | Rickettsiosis | Bovine cysticercosis | Mers-Cov |  | Cryptosporidiosis |
| 17 |  | E. coli | Taeniosis | Hydatidosis | Cyclosporiasis |  | Campylobacter |
| 18 |  | Trypanosoma | Sarcopsis | Porcine cysticercosis | Crimean-Congo hemorrhagic fever |  | Hepatitis E |
| 19 |  | Streptococcus suis | Cryptosporidiosis | Newcastle disease | Aspergillosis |  |  |
| 20 |  | Rift Valley Fever | Leishmaniasis | Orf (contagious ecthyma) | West Nile |  |  |

| **Disease search strings used in the systematic review of zoonoses in Somalia.** | |
| --- | --- |
| **Disease name** | **Search terms** |
| Anthrax | Anthrax OR malignant pustule OR malignant edema OR woolsorters disease OR woolsorter's disease OR Bacillus anthracis |
| Brucellosis | Brucellosis OR undulant fever OR malta fever OR Mediterranean fever OR brucella OR brucella abortus OR brucella melitensis |
| Bovine Tuberculosis | Bovine tuberculosis OR mycobacterium bovis OR zoonotic tuberculosis, Bovine TB |
| Rift Valley Fever | Rift Valley fever OR RVF OR RVF virus |
| Rabies | Rabies OR hydrophobia OR lyssa OR rabies virus |
| Echinococcosis | Echinococcosis OR hydatid cyst OR echinococcus granulosus OR hydatidosis |
| Q fever | Q fever OR Q-fever OR query disease OR coxiellosis OR coxiella burnetii |
| Salmonella | Salmonella OR salmonellosis OR salmonella infection OR non-typhoidal salmonella OR nontyphoidal salmonella |
| Taeniasis | Taeniasis OR taenia saginata OR beef tapeworm OR T. saginata OR Taeniarhynchus saginatus |
| Toxoplasma | Toxoplasma OR toxoplasmosis OR toxoplasma gondii |
| Schistosomiasis | Schistosomiasis OR snail fever OR bilharzia OR Bilharziasis OR blood-fluke disease OR Katayama fever |
| Zoonotic influenza viruses | Zoonotic influenza viruses OR avian influenza OR bird flu OR avian flu OR fowl plague |
| Trypanosomiasis | Trypanosomiasis OR trypanosomosis OR sleeping sickness OR Trypanosoma brucei rhodesiense |
| Leptospirosis | Leptospirosis OR weil disease OR weil's disease OR canicola fever OR hemorrhagic jaundice OR mud fever OR swineherd's disease OR leptospira |
| Leishmaniasis | Leishmaniasis OR kala azar OR kalaazar OR kala-azar |
| Cryptosporidiosis | Cryptosporidiosis OR crypto OR Cryptosporidium |
| Campylobacteriosis | Campylobacteriosis OR campylobacter OR campylobacter diarrhea OR campylobacter enteritis OR campylobacter intestinal infection |
| Hepatitis E | Hepatitis E OR hepatitis E virus |
| E coli | E. coli, Escherichia coli |
| Rickettsiosis | Rickettsiosis, Rickettsia |
| Staphylococcus aureus | Staphylococcus aureus, Staph aureus |
| Arbovirus | Arbovirus |
| Crimean-Congo haemorrhagic fever | Crimean-Congo haemorrhagic fever, CCHF, Crimean-Congo hemorrhagic fever orthonairovirus |
| West Nile Virus | West Nile virus, West Nile Fever |
| Liver fluke | Liver fluke, [Fasciolosis](https://en.wikipedia.org/wiki/Fasciolosis), Fasciola* |

WHO 2016. *Joint External Evaluation of IHR Core Capacities for the Republic of Somalia*,

Pieracci, E. G., Hall, A. J., Gharpure, R., Haile, A., Walelign, E.*, et al.* 2016. Prioritizing zoonotic diseases in Ethiopia using a one health approach. *One Health*, **2** 131-135.

Munyua, P., Bitek, A., Osoro, E., Pieracci, E. G., Muema, J.*, et al.* 2016. Prioritization of zoonotic diseases in Kenya, 2015. *PloS one*, **11** (8), e0161576.

Sekamatte, M., Krishnasamy, V., Bulage, L., Kihembo, C., Nantima, N.*, et al.* 2018. Multisectoral prioritization of zoonotic diseases in Uganda, 2017: A One Health perspective. *PloS one*, **13** (5), e0196799.

Kemunto, N., Mogoa, E., Osoro, E., Bitek, A., Kariuki Njenga, M.*, et al.* 2018. Zoonotic disease research in East Africa. *BMC infectious diseases*, **18** (1), 1-9.

Cavalerie, L., Wardeh, M., Lebrasseur, O., Nanyingi, M., McIntyre, K. M.*, et al.* 2021. One hundred years of zoonoses research in the Horn of Africa: A scoping review. *PLoS neglected tropical diseases*, **15** (7), e0009607.
